# Impact of childbirth history on dense breast in mammographic screening: a cross-sectional study

**DOI:** 10.1186/s12905-022-01772-4

**Published:** 2022-05-26

**Authors:** Tomohiro Ochi, Hiroko Tsunoda, Hideko Yamauchi, Osamu Takahashi

**Affiliations:** 1 Graduate School of Public Health, St. Luke’s International University, 10-1 Akashi-cho, Chuo-ku, Tokyo, 104-0044 Japan; 2 Department of Radiology, St. Luke’s International Hospital, Tokyo, Japan; 3 Department of Breast Surgical Oncology, St. Luke’s International Hospital, Tokyo, Japan; 4 Division of General Internal Medicine, Department of Internal Medicine, St. Luke’s International Hospital, Tokyo, Japan; 5 Department of Breast Surgery and Oncology, Nippon Medical School Hospital, Tokyo, Japan

**Keywords:** Parity, Dense breast, Mammographic screening, Breast neoplasms

## Abstract

**Background:**

The evaluation of breast density is important, because dense breast has been shown to be associated with increased risk of breast cancer and a greater risk of a false-negative diagnostic performance due to masking a tumor. Although the relationship between parity and dense breast is under investigation, conclusive evidence is lacking. We aimed to investigate whether parity affects breast density.

**Methods:**

The study design is a cross-sectional study. The subjects are healthy Japanese women who underwent opportunistic mammographic screening at the center for preventive medicine at a single institution from January 2016 to December 2018. Clinical characteristics and lifestyle factors were obtained from questionnaires. Breast density was categorized into 4 groups, namely, almost entirely fatty dense, scattered fibroglandular dense, heterogeneously dense, and extremely dense, according to the Breast Imaging Reporting and Data System. Heterogeneously and extremely dense were considered collectively as dense breast. Multivariate logistic regression analysis was conducted to investigate the relationship between parity and dense breast among premenopausal and postmenopausal women separately.

**Results:**

7612 premenopausal and 9252 postmenopausal women were investigated. Dense breast was shown in 62.6% of nulliparity, 57.3% of single parity, 47.3% of two parity, 37.6% of more than two parity among premenopausal women, and in 41.6% of nulliparity, 31.1% of single parity, 19.3% of two parity, 10.1% of more than two parity among postmenopausal women. For premenopausal women, two parity, single parity and nulliparity showed a higher risk for dense breast with statistically significance (Odds Ratio (OR) adjusted for potential confounding factors: 1.458 (95% Confidence interval (CI); 1.123–1.894), 2.349 (95%CI; 1.801–3.064), 3.222 (95%CI; 2.500–4.151), respectively), compared with more than two parity. For postmenopausal women, two parity, single parity and nulliparity had a higher risk (OR: 1.849 (95%CI; 1.479–2.312), 3.023 (95%CI; 2.385–3.830), 4.954 (95%CI; 3.975–6.174), respectively) with statistically significance, compared with more than two parity.

**Conclusions:**

Parity showed an inverse trend of having dense breast among both premenopausal and postmenopausal women. In particular, nulliparous women need to recognize their higher risk of dense breast. In the future, the declining fertility rate may affect the prevalence of dense breast in the world.

## Background

Dense breast on mammography is one of the most important risk factors for breast cancer, although many factors are considered to be related with the risk of breast cancer. Previous studies have shown that dense breast has been shown to have a 2–5 times higher risk of breast cancer [1–5]. To make matters worse, dense breast on mammography has been shown to have risks of masking breast cancer [6, 7]. The masking effect is one of the most important factors causing the reduced sensitivity in dense breasts, because the X-ray absorption coefficient of the mammary tissue in mammography is quite close to that of the breast cancer forming the mass. Thus, evaluation of breast density on mammography is important as a predictor of breast cancer risk and a masking risk estimator on screening.

The etiology of the dense breast is not definitively clear. Although the relationship between parity and dense breast is under investigation, many of these related studies are retrospective nature and involve relatively small sample [8–19]. Therefore, conclusive evidence regarding the association between a history of childbirth and mammographic density is lacking. Furthermore, as most studies were performed for white women, studies regarding with Asian women are fewer and most of them targeted relatively small sample [15–19]. Racial differences have been considered an important factor for breast density, and Asian women tend to have higher breast density than Caucasian [2, 20, 21]. It is unclear how the number of parity influences the breast density for Asian women including Japanese women. In this study, we aimed to investigate whether parous women have a lower mammographic density than nulliparous women on a large sample in Japan.

## Methods

### Study population

The subjects are general healthy women who underwent opportunistic mammographic screening for health check-up at the Center for Preventive Medicine at St. Luke’s International Hospital from January 2016 to December 2018. In Japan, many women undergo opportunistic mammographic screening periodically every one or two years. For women who revisited the screening several times in this period, only the data at the first visit was extracted, because the breast density does not change radically in three years during which data collection took place. Women with a history of breast cancer, women with any findings of mammography screening, and women without consent to this study were excluded.

### Design and setting

This was a cross-sectional study and the questionnaire collected demographic information concerning age, past medical history, current habit of alcohol consumption, which was chosen from three choices, none, occasional, or regular, according to frequency per week, smoking habit, which was chosen from three choices, never, past, or current, family history of breast cancer, parity, which was defined as the number of pregnancies that resulted in single or multiple childbirth except for stillbirth regardless of gestation period, menopausal status which was defined by the cessation of menstrual periods for one full year, and history of hormonal therapy use, including both premenopausal oral contraceptive use and postmenopausal hormone replacement therapy, which was selected from two choices, yes or no. Participants underwent physical measurements and mammography examination. Body mass index (BMI) was calculated as the body weight (kg) divided by square of height (m). The process of this study was to investigate and compare the characteristics of nulliparous women with that of parous women. The items of their characteristics compared in this study was selected, based on previous studies [8, 11, 16]. Then, the impact of each item for dense breast was calculated in the univariate analysis. When the items were considered statistically relevant (p < 0.25), those items were incorporated into the multivariate analysis as possible confounding factors. Then, we analyzed the impact of parity at the point of the risk for dense breast, adjusted by possible confounding factors. All analyses were carried out separately for premenopausal and postmenopausal women due to several reasons. First, mammographic density has been shown to be associated with menopausal status [10, 17, 22]. Second, previous studies have shown that the effects of several confounders can be different between premenopausal and postmenopausal women [9, 12, 16]. Third, some previous studies showed that the effect of childbirth for breast density was different by menopausal status [8, 12]. The results may advance our understanding of dense breast, and might eventually lead to better preventive strategies of breast cancer.

### Mammographic density

Digital mammography was taken with its quality certified by the Japan Central Organization on Quality Assurance of Breast Cancer (JCOQABC). Mammograms were taken in two directions, mediolateral oblique and craniocaudal view. Breast density was categorized into 4 groups, namely, almost entirely fatty dense, scattered fibroglandular dense, heterogeneously dense, and extremely dense, according to the Breast Imaging Reporting and Data System (BI-RADS) [23]. Heterogeneously dense and extremely dense breast categories were considered collectively as dense breast. These breast densities were evaluated by JCOQABC authorized physicians.

## Statistical analysis

We constructed contingency tables of the clinical features and compared the characteristics between parous women and nulliparous women. The independent t-test was used for comparison of quantitative variables and the chi-square test was used for comparisons of categorical variables. The univariate logistic regression model was applied to analyze the odds ratio (OR) of dense breast with 95% confidence interval (CI). To adjust the effects of possible confounders of statistical significance in univariate analysis, the multivariate analysis was performed to calculate the adjusted OR. Statistical significance was assessed, with a *p*-value cut-off point at 0.05 in all analyses. All statistical analyses were performed using SPSS version 24.0 (SPSS, Inc., Chicago, IL, USA).

## Results

### Characteristics

The characteristics of the women recruited in this study are summarized in Table 1. For 7612 premenopausal women, mean age was 44.58, mean BMI was 21.34, the number of parity was 0 for 3878 (50.9%), 1 for 1604 (21.1%), 2 for 1800 (23.6%), and ≥ 3 for 330 (4.3%). About the composition of mammographic density categories, almost entirely fatty dense accounted for 1.6% (n = 122), scattered fibroglandular dense for 41.6% (n = 3168), heterogeneously dense for 54.2% (n = 4129), and extremely dense for 2.5% (n = 193). Dense breast represented a total of 56.7% (i.e., 54.2 + 2.5%). In contrast, for 9252 postmenopausal women, mean age was 61.51, mean BMI was 21.65, the number of parity was 0 for 3279 (35.4%), 1 for 1548 (16.7%), 2 for 3309 (35.8%), and ≥ 3 for 1116 (12.0%). About breast density, almost entirely fatty dense accounted for 6.6% (n = 615), scattered fibroglandular dense for 65.3% (n = 6039), heterogeneously dense for 27.3% (n = 2530), and extremely dense for 0.7% (n = 68). Dense breast represented a total of 28.0% (i.e., 27.3 + 0.7%). Premenopausal women tended to have higher breast density than postmenopausal women like previous studies [10, 17, 22].

**Table 1:** Baseline characteristics of premenopausal and postmenopausal women. The characteristics of the women recruited in this study were investigated for both premenopausal and postmenopausal women separately

	Premenopausal women (n = 7612)	Postmenopausal women (n = 9252)
Age mean (SD)	44.58 (5.54)	61.51 (8.37)
BMI mean (SD)	21.34 (3.26)	21.65 (3.42)
Parity		
0	3878 (50.9%)	3279 (35.4%)
1	1604 (21.1%)	1548 (16.7%)
2	1800 (23.6%)	3309 (35.8%)
≥ 3	330 (4.3%)	1116 (12.0%)
Smoking status		
Never	6191 (81.3%)	7685 (83.1%)
Past	1054 (13.8%)	1245 (13.5%)
Current	367 (4.8%)	322 (3.5%)
Alcohol status		
None	3505 (46.0%)	5305 (57.3%)
Occasional	1184 (15.6%)	1237 (13.4%)
Regular	2923 (38.4%)	2710 (29.3%)
Family history of breast cancer	1487 (19.5%)	1605 (17.3%)
Hormonal therapy	1091 (14.3%)	1301 (14.1%)
Breast density		
Almost entirely fatty dense	122 (1.6%)	615 (6.6%)
Scattered fibroglandular dense	3168 (41.6%)	6039 (65.3%)
Heterogeneously dense	4129 (54.2%)	2530 (27.3%)
Extremely dense	193 (2.5%)	68 (0.7%)

Premenopausal women comprised 3878 nulliparous and 3734 parous women and their demographic characteristics are shown in Table 2. Parous women showed significantly higher age (nulliparous; 44.12 ± 5.95 years old, parous; 45.06 ± 5.04 years old), and lower BMI (nulliparous; 21.51 ± 3.45 kg/m^2^, parous; 21.17 ± 3.03 kg/m^2^). Parous women showed less likely to smoke (nulliparous; 6.8%, parous; 2.8%), less likely to drink (nulliparous; 41.5%, parous; 35.1%), and more likely to have hormonal therapy (nulliparous; 13.8%, parous; 15.8%). In contrast, postmenopausal women comprised 3279 nulliparous and 5973 parous women and their demographic characteristics are shown in Table 2. Parous women showed significantly higher age (nulliparous; 58.93 ± 7.57 years old, parous; 62.92 ± 8.45 years old), and higher BMI (nulliparous; 21.56 ± 3.65 kg/m^2^, parous; 21.70 ± 3.29 kg/m^2^). Parous women were less likely to smoke (nulliparous, 5.3%; parous, 2.5%), less likely to drink (nulliparous, 35.3%; parous, 26.0%), and less likely to have hormonal therapy (nulliparous, 18.6%; parous, 12.5%).

**Table 2:** Differences in the characteristics of nulliparous and parous women. The differences in the characteristics of nulliparous and parous women were investigated for both premenopausal and postmenopausal women separately. For premenopausal women, parous women showed higher age and lower BMI. Parous women showed less likely to smoke and less likely to drink, and were more likely to have hormonal therapy. In contrast, for postmenopausal women, parous women showed higher age and higher BMI. Parous women showed less likely to smoke and less likely to drink and less likely to have hormonal therapy

	Premenopausal women (n = 7612)			Postmenopausal women (n = 9252)		
	Nulliparous (n = 3878)	Parous (n = 3734)	*p*	Nulliparous (n = 3279)	Parous (n = 5973)	*p*
Age mean (SD)	44.12 (5.95)	45.06 (5.04)	< 0.001	58.93 (7.57)	62.92 (8.45)	< 0.001
BMI mean (SD)	21.51 (3.45)	21.17 (3.03)	< 0.001	21.56 (3.65)	21.70 (3.29)	< 0.001
Smoking status			< 0.001			< 0.001
Never	3079 (79.4%)	3112 (83.3%)		2564 (78.2%)	5121 (85.7%)	
Past	537 (13.8%)	517 (13.8%)		541 (16.5%)	704 (11.8%)	
Current	262 (6.8%)	105 (2.8%)		174 (5.3%)	148 (2.5%)	
Alcohol status			< 0.001			< 0.001
None	1627 (42.0%)	1878 (50.3%)		1704 (52.0%)	3601 (60.3%)	
Occasional	640 (16.5%)	544 (14.6%)		418 (12.7%)	819 (13.7%)	
Regular	1611 (41.5%)	1312 (35.1%)		1157 (35.3%)	1553 (26.0%)	
Family history of breast cancer	772 (19.9%)	715 (19.1%)	0.404	596 (18.2%)	1009 (16.9%)	0.119
Hormonal therapy	515 (13.8%)	576 (15.8%)	0.017	586 (18.6%)	715 (12.5%)	< 0.001

### Relationship between number of parity and dense breast

**Fig. 1:**
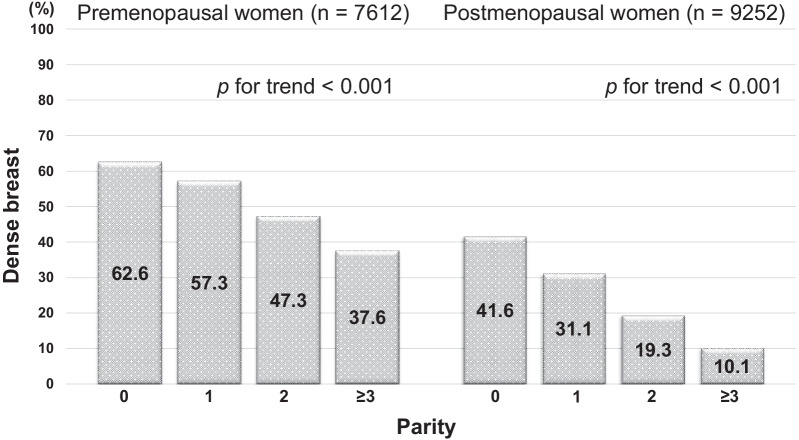
Relationship between parity and dense breast. For premenopausal women, dense breast was shown among 62.6% of nulliparous women, and 57.3% of women with single parity, 47.3% for women with two parity, and 37.6% for women with more than two parity. In contrast, for postmenopausal women, dense breast was shown among 41.6% of nulliparous women, and 31.1% of women with single parity, 19.3% for women with two parity, and 10.1% for women with more than two parity

The relationship between the number of parity and dense breast is shown in Fig. 1. Among premenopausal women, the prevalence of dense breast was 62.6% for nulliparous women, 57.3% for women with single parity, 47.3% for women with two parity, and 37.6% for women with more than two parity. The higher the number of parity, the lower the prevalence of dense breast. The difference in the prevalence of dense breast according to the number of parity was significant (*p* for trend < 0.001). Among postmenopausal women, a similar trend was shown. The prevalence of dense breast was 41.6% for nulliparous women, 31.1% for women with single parity, 19.3% for women with two parity, and 10.1% for women with more than two parity. The higher the number of parity, the lower the prevalence of dense breast. The difference in the prevalence according to the number of parity was significant (*p* for trend < 0.001).

### Odds ratio for dense breast

For premenopausal women, two parity, single parity, and nulliparity had a higher risk of dense breast (OR: 1.490 (95% CI; 1.170–1.8196), 2.229 (95% CI; 1.747–2.844), 2.782 (95% CI; 2.206–3.508), respectively), compared with women with more than two parity. In the multivariate analysis, adjusted by age, BMI, smoking status and alcohol status, parity still showed a significant relationship. Compared with women with more than two parity, two parity and single parity showed a higher risk of dense breast (OR: 1.458 (95% CI; 1.123–1.894), OR: 2.349 (95% CI; 1.801–3.064), respectively), and nulliparity showed a further higher risk of dense breast (OR; 3.222 (95% CI; 2.500–4.151)) (Table 3).

**Table 3:** Odds ratio for dense breast among premenopausal women. In the multivariate analysis, adjusted by age, BMI, and alcohol status, parity still showed a significant relationship. Compared with women with more than two parity, two parity, single parity, nulliparity showed a higher risk of dense breast (OR: 1.458 (95% CI; 1.123–1.894), OR: 2.349 (95% CI; 1.801–3.064), OR: 3.222 (95% CI; 2.500–4.151), respectively)

	Univariate	*p*	Multivariate	*p*
Age	0.941 (0.933–0.949)	< 0.001	0.958 (0.949–0.967)	< 0.001
BMI	0.756 (0.742–0.770)	< 0.001	0.756 (0.742–0.771)	< 0.001
Parity				
0	2.782 (2.206–3.508)	< 0.001	3.222 (2.500–4.151)	< 0.001
1	2.229 (1.747–2.844)	< 0.001	2.349 (1.801–3.064)	< 0.001
2	1.490 (1.170–1.896)	< 0.001	1.458 (1.123–1.894)	0.005
≥ 3 (ref)	–	–	–	–
Trend*	0.728 (0.693–0.764)	< 0.001	0.676 (0.640–0.714)	< 0.001
Smoking status				
Never (ref)	–	–	–	–
Past	1.001 (0.877–1.142)	0.991	0.969 (0.862–1.115)	0.592
Current	0.828 (0.670–1.022)	0.079	0.699 (0.549–0.889)	0.003
Alcohol status				
Non (ref)	–	–	–	–
Occasional	1.091 (0.955–1.246)	0.199	1.000 (0.862–1.160)	0.998
Regular	1.205 (1.091–1.330)	< 0.001	1.097 (0.981–1.227)	0.106
Family history of breast cancer	1.006 (0.897–1.128)	0.921	–	–
Hormonal therapy	0.925 (0.813–1.053)	0.238	0.937 (0.812–1.082)	0.378

*To calculate *p* for trend, variables were modeled as continuous value

In contrast, for postmenopausal women, two parity, single parity and nulliparity had a higher risk of dense breast (OR: 2.124 (95% CI; 1.717–2.628), 4.013 (95% CI; 3.214–5.012), 6.322 (95% CI; 5.143–7.772), respectively), compared with women with more than two parity. In the multivariate analysis, adjusted by age, BMI, smoking status, alcohol status, and hormonal therapy, parity still showed a significant relationship. Compared with women with more than two parity, two parity and single parity had a higher risk of dense breast (OR: 1.849 (95% CI; 1.479–2.312), OR: 3.023 (95% CI; 2.385–3.830), respectively), nulliparity showed a further higher risk of dense breast (OR: 4.954 (95% CI; 3.975–6.174)) (Table 4).

**Table 4:** Odds ratio for dense breast among postmenopausal women. In the multivariate analysis, adjusted by age, BMI, smoking status, alcohol status, and hormonal therapy, parity still showed a significant relationship. Compared with women with more than two parity, two parity, single parity and nulliparity had a higher risk of dense breast (OR: 1.849 (95% CI; 1.479–2.312), OR: 3.023 (95% CI; 2.385–3.830), OR: 4.954 (95% CI; 3.975–6.174), respectively)

	Univariate	*p*	Multivariate	*p*
Age	0.941 (0.933–0.947)	< 0.001	0.959 (0.952–0.966)	< 0.001
BMI	0.751 (0.736–0.765)	< 0.001	0.753 (0.737–0.768)	< 0.001
Parity				
0	6.322 (5.143–7.772)	< 0.001	4.954 (3.975–6.174)	< 0.001
1	4.013 (3.214–5.012)	< 0.001	3.023 (2.385–3.830)	< 0.001
2	2.124 (1.717–2.628)	< 0.001	1.849 (1.479–2.312)	< 0.001
≥ 3 (ref)	–	–	–	–
Trend*	0.565 (0.539–0.592)	< 0.001	0.575 (0.543–0.610)	< 0.001
Smoking status				
Never (ref)	–	–	–	–
Past	1.122 (0.984–1.280)	0.085	1.040 (0.894–1.209)	0.612
Current	1.385 (1.095–1.753)	0.007	0.946 (0.723–1.238)	0.684
Alcohol status				
Non (ref)	–	–	–	–
Occasional	1.320 (1.150–1.514)	< 0.001	1.320 (1.129–1.542)	< 0.001
Regular	1.757 (1.589–1.943)	< 0.001	1.250 (1.113–1.404)	< 0.001
Family history of breast cancer	1.031 (0.915–1.162)	0.612	–	–
Hormonal therapy	1.355 (1.195–1.536)	< 0.001	0.975 (0.846–1.123)	0.722

*To calculate *p* for trend, variables were modeled as continuous value

## Discussion

In this cross-sectional study, we found that nulliparous women had a higher risk of dense breast than parous women and that the number of parity was inversely associated with the prevalence of dense breast for both premenopausal and postmenopausal women. It appeared that the smaller the number of parity, the higher the prevalence of dense breast. Among previous studies which investigated the relationship between parity and breast density separately by menopausal status, some studies showed that the number of parity was significantly associated with a reduction in mammographic density in contrast with nulliparous women among both premenopausal and postmenopausal women [10, 12, 13]. Other studies showed statistically significant results only for postmenopausal women, but not for premenopausal women [8, 16]. Although any results did not show the opposite direction that the larger number of parity caused dense breast, the impact of childbirth seemed various according to each study. This is considered to be caused by several reasons. It might be due to different type of study design, different outcome measurement index of dense breast like quantitative or qualitative way, different categorization of parity like dichotomous or continuous index, different type of sample population at the point of race, age. And small sample size might decrease the statistical power. To the best of our knowledge, this is the largest study for Asian women, and our results could lead a statistically significant findings among both premenopausal and postmenopausal women, and may advance the present knowledge on the association between parity and breast density for the Asian women. Although the biological mechanism of parity toward breast density is not clearly shown, a previous study hypothesized that the mammary gland in parous women probably contains epithelial cells that are more differentiated, less proliferative, and less susceptible to transformation [24].

Dense breast on mammography comprises the risk of a false-negative finding owing to the masking effect of dense tissue, which may hide breast cancer [6, 7]. The sensitivity of mammography is reduced in women with dense breasts [25]. In a previous study, the sensitivity of mammography screening was reported to be 90.7% for fatty breast, 78.9% for scattered dense breast, 68.3% for heterogeneously dense breast, and 51.0% for extremely dense breast [26]. The higher the breast density, the lower the sensitivity. To supplement this issue, there are immense discussions about screening system. The supplemental use of other modalities such as ultrasonography, magnetic resonance imaging (MRI), digital breast tomosynthesis (DBT), and the interval of screening test have been under consideration. Although some of them were reported to be effective in detecting early-stage, breast cancers [27–29], studies have not evaluated whether supplemental imaging reduces advanced breast cancer rates or breast cancer mortality. There is insufficient evidence on whether additional imaging is effective as a mass screening tool [30]. To make matters worse, there is a risk that additional modality will result in even greater disadvantages of higher recall and biopsy rates [28]. In any case, mammography is the only method of breast cancer screening that has been proven to reduce mortality at present. Regarding the screening interval, a large cohort study examined the proportion of advanced breast cancers by screening mammography frequency and found that the proportion of advanced tumors was greater for biennial than annual screening for women aged 40–49 years with extremely dense breasts, but similar for women aged from 50 to 74 [31]. Annual screening for women with dense breasts could result in more harm than benefit with increasing risk of anxiety, benign breast biopsies, and overdiagnosis [30]. Women and health care providers should discuss breast density in the context of the decline of mortality rate on mass screening.

As a strategic health promotion program, the concept of breast awareness is recommended to be introduced [32]. The basic concept is for the general women to be familiar with the normal condition and feel of their own breasts. Furthermore, women should attend breast screenings from the specified age. Therefore, clinicians can provide more effective and clinically applicable health education by making women aware that mammograms are more likely to be dense in women who have given birth infrequently, informing them of the importance of breast awareness, and encouraging them to check their own breasts and inform their doctors of any changes in their breasts. Tailor-made screening system adopting other kinds of screening modality or adapting interval period based on each person’s risk can bring more profit from a micro perspective, because the benefit of mammography is not equal for all women. However, it is not clear whether this system will provide larger beneficial effects over its risk of overdiagnosis at present. Further research to evaluate the balance of risk and benefit is necessary.

We believe that this study could construct robust evidence. The subjects of this study were recruited from a general screening program, and a large sample size, as much as 16,864 women were investigated. This study will be helpful in tackling upcoming global trends in declining birthrate. The total fertility rate (TFR), which is defined as the number of children who would be born per woman, has been decreasing globally from 4.7 in 1950 to 2.4 in 2017. This means about 49.4% decline in TFR observed in this period [33]. Furthermore, TFR was forecasted to keep declining, and will reach 1.66 in 2100. About 183 countries including not only developed countries but also developing countries were forecasted to have a TFR lower than 2.1, which is considered the minimum rate necessary for generational replacement of the population [34]. In the future, the declining fertility rate may have a risk of affecting the prevalence of dense breast in the world.

Our study has several potential limitations. First, this study was performed using a cross-sectional design and did not allow us to research temporal associations. Further study with a follow-up survey for these population may acquire additional information regarding the relationship of parity with breast cancer incidence and subtype. Second, recall bias may potentially existed, because most of the demographic data were extracted from the questionnaires. Third, there may be other suspicious confounders, that affect the outcome including breastfeeding, age at first birth, miscarriage. In particularly, breastfeeding has a strong relationship with parity, and affects the endogenous hormonal level. Although previous studies have investigated the impact of breastfeeding on breast density, the association between the duration of breastfeeding and mammographic breast density had discrepant results [12, 13]. Although the duration and type of breastfeeding was thought to affect the results, a fixed form of questionnaire failed to collect data regarding breastfeeding and could not be taken into consideration in our current study. Fourth, smoking and drinking status were incorporated in the multivariate analysis as confounding factors, although they might play a role as mediation effects rather than confounding factors. This might lead to statistical over-adjustment and different outcome.

## Conclusions

Parity showed an inverse trend of having dense breast among both premenopausal and postmenopausal Asian women including Japanese. As the number of parity decreases, the more likely women appeared to have dense breast on mammography. In particular, nulliparous women need to be more aware of their own breast because of their higher risk of dense breast. Our findings significantly added novel information regarding the relationship between parity and breast density to the present knowledge of dense breast.

## Data Availability

The datasets generated and/or analyzed during the current study are available from the corresponding author on reasonable request.

## Abbreviations

BMI: Body mass index
JCOQABC: Japan Central Organization on Quality Assurance of Breast Cancer
BI-RADS: Breast imaging reporting and data system
OR: Odds ratio
95% CI: 95% Confidence interval
